# Assessment of Schlemm’s canal with swept-source optical coherence tomography in Graves’ ophthalmopathy

**DOI:** 10.1007/s00417-024-06397-x

**Published:** 2024-02-24

**Authors:** Raziye Dönmez Gün, Titap Yazıcıoğlu, Murat Oklar, Naile Gökkaya

**Affiliations:** 1 Department of Ophthalmology, Istanbul Kartal Doctor Lütfi Kırdar City Hospital, Semsi Denizer Street, E-5 Kartal, 34890 Istanbul, Turkey; 2 Department of Endocrinology and Metabolism, Istanbul Kartal Doctor Lütfi Kırdar City Hospital, Istanbul, Turkey

**Keywords:** Schlemm’s canal, Swept-source optical coherence tomography, Graves’ ophthalmopathy, Glaucoma

## Abstract

**Purpose:**

To evaluate the Schlemm’s canal (SC) parameters obtained by swept-source optical coherence tomography (OCT) different in Graves’ ophthalmopathy (GO) eyes compared to healthy eyes.

**Methods:**

This cross-sectional observational study evaluated 64 eyes of 32 GO cases and 56 eyes of 28 healthy controls. The study was conducted between October 2020 and June 2021. SC images were obtained from the temporal limbus of individuals using swept-source OCT. SC length (SCL) and SC area (SCA) were measured. The relationship between SC parameters in the patient group and intraocular pressure (IOP), retinal nerve fiber layer (RNFL) thickness, Graves’ disease (GD) duration, and clinical activity score (CAS) was evaluated.

**Results:**

In the GO group, 64 eyes of 32 patients were evaluated, and in the age and gender-matched healthy control group, 56 eyes of 28 individuals were assessed. SC images from 4 eyes of 4 patients in the patient group and 1 eye of 1 patient in the control group were not clear, preventing SCL and SCA measurements for these eyes. SCL and SCA measurements were found to be lower, and IOP and Hertel values were higher in the GO group compared to the healthy controls. However, no significant correlation was observed between SCL and SCA with IOP, RNFL thickness, GD duration, GO duration, or CAS in the GO group. In the GO group, the mean value of SCA was found to be higher in eyes with glaucoma or OHT compared to those without.

**Conclusion:**

These findings indicate that SC in GO-affected eyes is shorter and has a smaller area than in healthy individuals. Additionally, higher IOP and Hertel values were observed in the GO group compared to healthy controls. This study suggests that assessing SC using anterior segment OCT could provide valuable insights into the regulation of IOP and the development of glaucoma in GO-affected eyes.


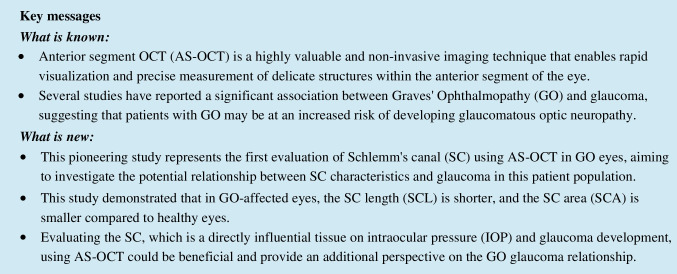


## Introductıon

GO is a complex autoimmune orbitopathy that constitutes the ophthalmic manifestations of thyroid-associated autoimmune disease. It is the most common extrathyroidal expression of GD. GO can cause retracting eyelids, proptosis, double vision, edema, and vision loss [1]. It affects orbital fat tissue, extraocular muscles, eyeballs, and eye appendages [2]. Some studies have shown that individuals with thyroid disease have higher intraocular pressure (IOP) and an increased risk of glaucoma [3, 4]. Cockerham reported that a prolonged duration of active GO is associated with the progression of glaucomatous damage [4]. Elevated IOP in GO can be attributed to various reasons. One of them is inflammation-related swelling in extraocular muscles, leading to restriction and compression of the eyeball [5]. Another reason could be a decrease in orbital venous drainage, resulting in increased episcleral venous pressure [6]. Furthermore, the capacity of human trabecular meshwork tissue to respond to thyroid hormones has been suggested as another contributing factor [7]. Additionally, abnormal accumulation of glycosaminoglycans in the trabecular meshwork and SC could lead to increased outflow resistance [8]. Despite efforts to explain the increased IOP in GO through these different mechanisms, the relationship between GO and IOP still remains enigmatic.

In the human eye, IOP is regulated, in part, by the outflow of aqueous humor through the trabecular meshwork, which then drains into SC before reaching the collector channels and ultimately emptying into the scleral veins [9]. Previous research has suggested that individuals with glaucoma may have a smaller SC compared to healthy individuals. The researchers hypothesized that a reduced SC size could be associated with elevated IOP, as SC size is known to be related to outflow facility [10]. Several studies have evaluated SC parameters in eyes with glaucoma [9–11]. Additionally, the relationship between GO and glaucoma has been demonstrated in various studies [12–14].

In individuals with GO, the non-invasive measurement of SCL and SCA could offer a valuable tool for both clinical and basic research, as well as the clinical management of potential glaucoma and ocular hypertension (OHT). The aim of our study is to evaluate SCL and SCA in GO-affected eyes and compare them with healthy controls. Additionally, we explored whether these parameters in the GO group are associated with IOP, GD duration, GO duration, and CAS. Finally, SC measurements of eyes without glaucoma or OHT in patients with Graves’ ophthalmopathy were compared with those of eyes with glaucoma or OHT. To the best of our knowledge, this is the first in vivo study that evaluates SC parameters in patients with GO using anterior segment swept-source OCT and examines the GO-IOP relationship from this perspective.

## Methods

### Study design and participants

This cross-sectional study was conducted at Dr. Lütfi Kırdar City Hospital, involving the Department of Ophthalmology and Division of Adult Endocrinology, between October 2020 and June 2021. The study received approval from the hospital ethics committee (decision number: 2022/514/224/6) and was carried out following the principles of the Declaration of Helsinki. Written informed consent was obtained from all participants, including 64 eyes of 32 individuals diagnosed with GO (10 male, 22 female) who were regularly monitored at our hospital’s ophthalmology clinic, and 56 eyes of 28 healthy control patients (9 male, 19 female) who were evaluated at the outpatient clinic of the Department of Ophthalmology. GO-affected individuals were evaluated according to the European Group on Graves’ Orbitopathy (EUGOGO) study criteria. The eyes of patients with GO were divided into two groups: those without glaucoma or OHT and those with glaucoma or OHT.

### Clinical evaluation

Each participant underwent a detailed ophthalmic examination, which included an assessment of best-corrected visual acuity (BCVA) using Snellen chart and conversion to logMAR equivalent, IOP measurements in the primary position using Goldmann applanation tonometry, slit-lamp biomicroscopy, ophthalmoscopy (dilated examination using a 90-dioptre lens), gonioscopy (with Zeiss Four-mirror lens), Hertel exophthalmometry measurements in the primary position, and CAS classification of GO. Central corneal thickness (CCT) measurements were obtained using a Scheimpflug camera (Sirius Topography Device, CSO, Frenze, Italy), and anterior segment images for SC evaluation and RNFL thickness measurements were obtained using swept-source OCT (Triton DRI-OCT, Topcon, Tokyo, Japan). All selected OCT images were exported in JPEG format and imported into the ImageJ program (ImageJ 1.54d, National Institutes of Health) to enable measurement. Patients diagnosed with glaucoma or ocular hypertension during follow-up visits were noted.

### Exclusion criteria

Patients with spherical refractive error over 3 diopters or astigmatism over 2 diopters, known additional ocular diseases, a history of ocular surgery or laser treatment, or a history of ocular trauma were excluded from both the GO and control groups. In addition, healthy control patients were excluded if they had a history of known systemic diseases. Patients in the GO group receiving systemic steroid treatment were also excluded from the study.

### Data collection

Examinations and measurements of all participants were conducted between 08:30 and 09:30 AM.

SC imaging was performed using swept-source optical coherence tomography (OCT), and the calculation of SC measurements was performed using Image J.

AS-OCT is a form of OCT technology used for clinical evaluation and scientific investigation of ocular surface and anterior segment diseases [15–17]. SC imaging was performed using swept-source optical OCT with the Triton DRI-OCT device (Topcon, Tokyo, Japan), which features an anterior segment adapter. The OCT system uses a 1050-nm light source with a scan rate of 100 kHz and a scan depth of 3 mm [18].

**Fig. 1:**
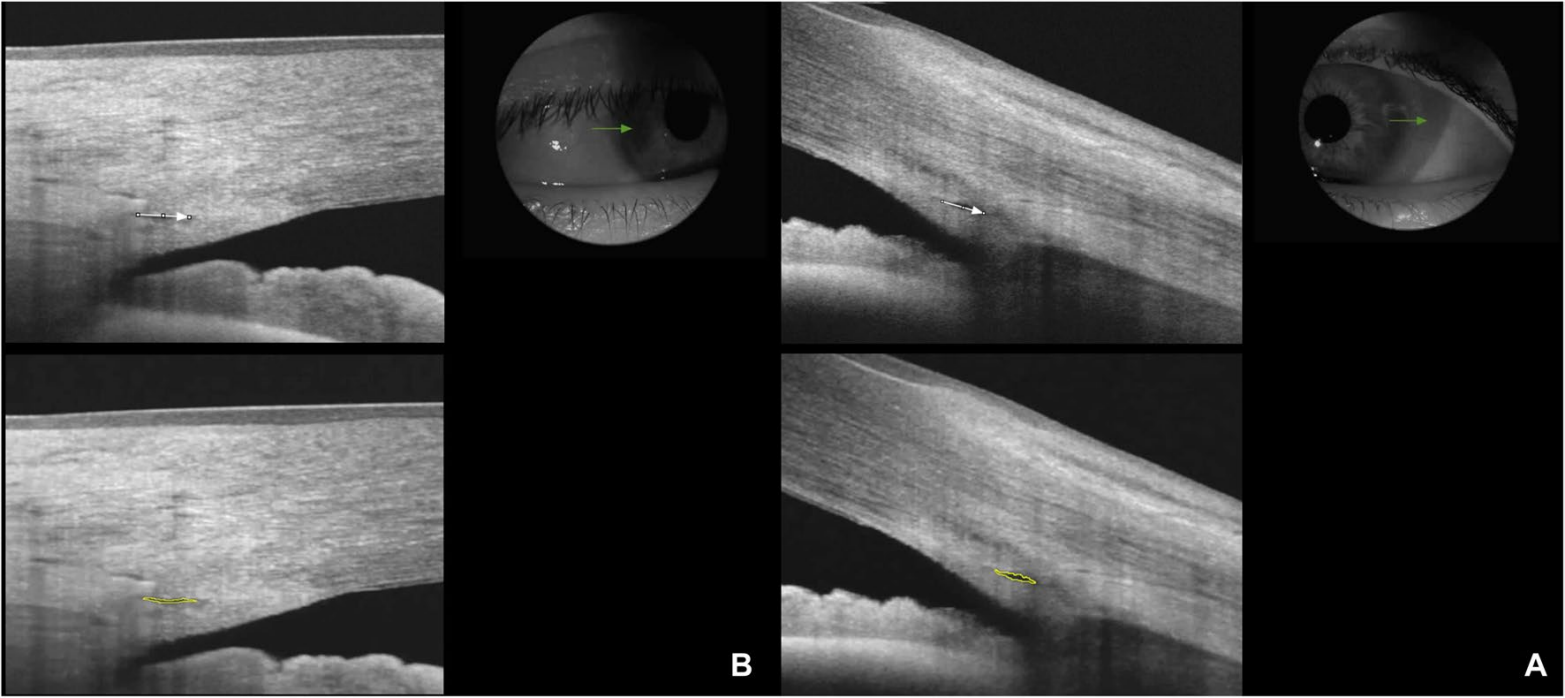
Schlemm’s canal images captured using anterior segment capture mode: (**1A**) a patient’s left eye and (**1B**) a healthy control’s right eye

After mounting the anterior segment adapter to the OCT device, SC images were obtained by an experienced investigator (RDG) under dark room conditions before pupil dilation. The temporal limbus was imaged, and the fixation was adjusted to the nasal area. SC images were captured using the Line(H) Anterior seg. 3.0 mm capture mode for the left eye at the 3 o’clock position and for the right eye at the 9 o’clock position (Fig. 1A). Patients were asked to open their eyes as wide as possible during the examination. Gentle assistance was provided by the examiner, using their fingers to keep the eyelids open without applying pressure to the eyeball. An eye speculum was not used to keep the eyes open. Three consecutive images of SC were obtained.

Two experienced investigators (RDG and MO), who were blinded to the subject groups, evaluated the images. All selected images were exported in JPEG format and imported into ImageJ software (http://imagej.nih.gov/ij/; provided in the public domain by the National Institutes of Health, Bethesda, MD, USA) for measurement [19]. SCL was defined as the axial length of the thin, black, and lucent space. The SCA was depicted as the area surrounded by the outline of SC [9]. The SCL and SCA were measured manually by two researchers (RDG and MO) using only images in which SC was completely observable (Fig. 1B). SCL was defined as the average of three measurements of its axial length.

### Statistical analysis

Descriptive statistics, including mean, standard deviation, minimum, median, and maximum, were used to describe continuous variables. The comparison of two variables that were independent and did not show a normal distribution was conducted using the Mann–Whitney U test. The comparison of two variables that were independent and showed a normal distribution was performed using the Student *t*-test. The correlation analysis of two variables that did not exhibit normal distribution was conducted using Spearman’s rank correlation test. The statistical significance level was set at 0.05. The analyses were performed using SPSS version 24 software.

## Results

A total of 120 eyes from 60 subjects were included in the study, consisting of 64 eyes from 32 patients with GO and 56 eyes from 28 age- and sex-matched normal controls. The characteristics of the study subjects are presented in Table 1. In 4 eyes of 4 patients from the GO group and 1 eye of 1 patient from the control group, clear images could not be obtained using anterior segment OCT, and thus, measurements of SCL and SCA were not possible for these eyes.

**Table 1:** Demographic characteristics of the ıncluded groups and ınter-group comparisons

Mean ± SD Median (min.–max.)		Control		Patient		*p*
Age (year)		40 ± 12 42– (19–60)		40 ± 14 41– (19–69)		0,960
SCL (µm)		289 ± 50 295– (153–413)		259 ± 54 257– (130–371)		**0,003**
SCA (µm^2^)		5703 ± 2146 5521– (2176–11,367)		4628 ± 1997 4238– (1916–9066)		**0,006**
IOP (mmHg)		16 ± 2 16 (10–20)		17 ± 3 18 (12–24)		< 0,001*
CCT (µm)		545 ± 31 546– (480–627)		544 ± 33 546– (485–650)		0,897
C/D		0,3 ± 0,2 0,4– (0–0,6)		0,2 ± 0,2 0,3– (0–0,6)		0,004*
Hertel values (mm)		16 ± 1 16– (14–19)		19 ± 3 18– (13–25)		** < 0,001**
Mean RNFL (µm)		110 ± 8 108– (97–129)		108 ± 10 107– (87–136)		0,320
		***N* (%)**		***N* (%)**		p**
Gender	Female	19	67,9%	22	68,8%	1,000
	Male	9	32,1%	10	31,3%	

Student t-test, Mann–Whitney U test*, Chi-square test**

*SD*, standard deviation; *SCL*, Schlemm’s canal length; *SCA*, Schlemm’s canal area; *IOP*, intraocular pressure; *CCT*, central corneal thickness; *C/D*, cup to disc; *RNFL*, retinal nerve fiber layer

*p* < 0.05 was shown in bold

**Fig. 2:**
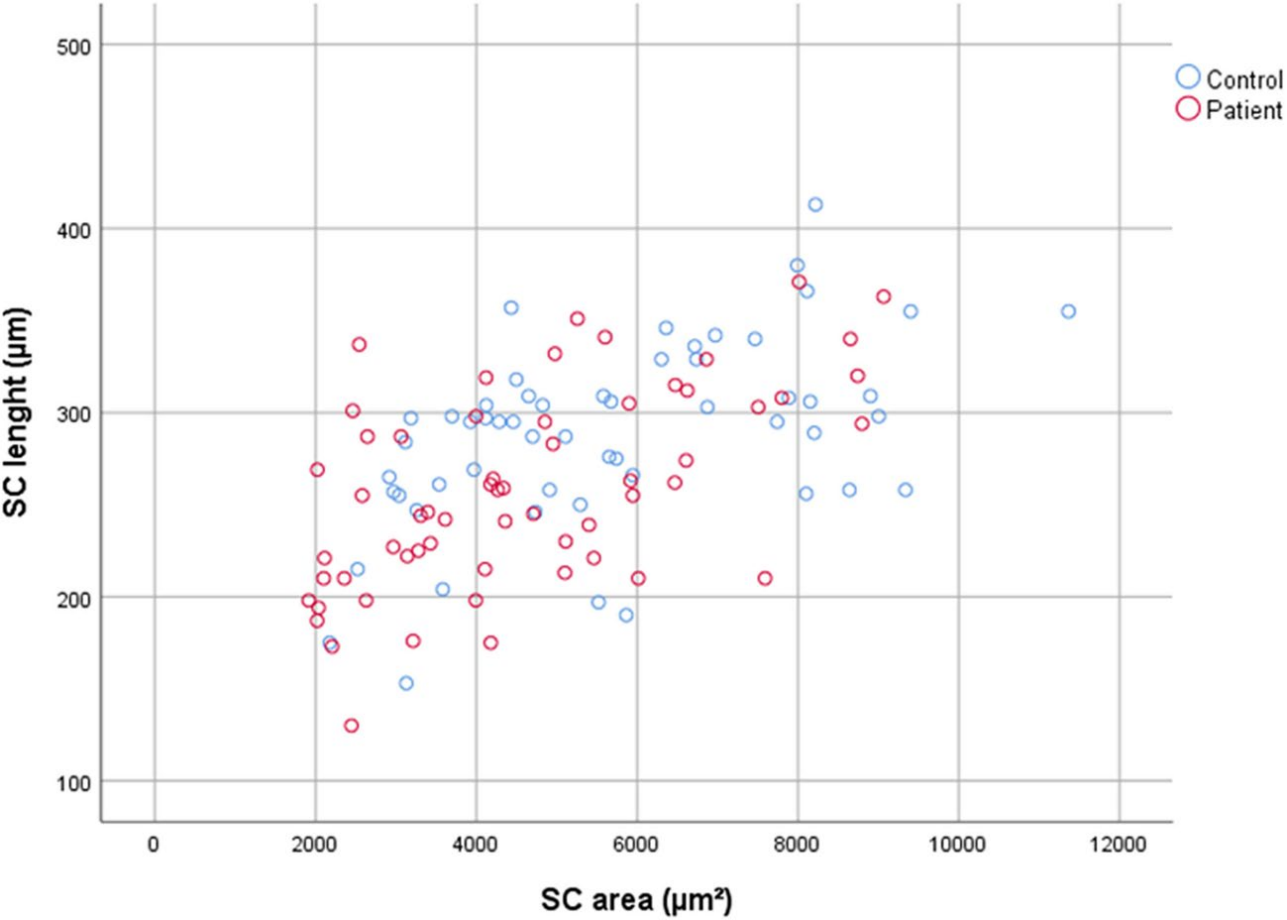
The scatter plot of SCL and SCA between the control and patient groups

In the GO group, SCL and SCA were found to be significantly lower (*p* = 0.003 and *p* = 0.006, respectively), while the mean IOP and Hertel values were significantly higher (*p* < 0.001 and *p* < 0.001, respectively) compared to the control group (Table 1). The scatter plot of SCL and SCA between the control and patient groups is shown in Fig. 2.

No statistically significant correlation was found between SCL, SCA, IOP, and RNFL values in the GO group (*p* > 0.05). Similarly, no statistically significant correlation was observed between SCL, SCA, and GD duration, GO duration, and CAS in the GO group (*p* > 0.05) (Table 2).

**Table 2:** Correlation analysis of SCL and SCA with IOP and RNFL in the patient group

Mean ± SD Median (min.–max.)		IOP	Mean RNFL*	GD duration	GO duration *	CAS
SCL	***r***	0,152	− 0,117	− 0,118	− 0,135	− 0,187
	***p***	0,246	0,375	0,565	0,511	0,349
SCA	***r***	0,163	− 0,034	0,361	− 0,004	− 0,259
	***p***	0,213	0,796	0,070	0,983	0,192

Pearson, Spearman’s rho correlation*

*SCL*, Schlemm’s canal length; *SCA*, Schlemm’s canal area; *IOP*, intraocular pressure; *RNFL*, retinal nerve fiber layer; *SD*, standard deviation; *GD*, Graves’ disease; *GO*, Graves’ ophthalmopathy; *CAS*, clinically activity score

In both groups, all participants reported no family history of glaucoma. During follow-up, 2 eyes (3.1%) in the GO group were diagnosed with glaucoma, and 8 eyes (12.5%) were diagnosed with ocular hypertension. These patients are currently under regular follow-up at our clinic. None of the patients in the control group received a diagnosis of glaucoma or ocular hypertension. In eyes with GO, SCA was found to be higher in the group with glaucoma or OHT compared to those without (*p* < 0.05) (Table 3).

**Table 3:** Subgroup comparison of SCL and SCA in GO patients

Mean ± SD Median (min.–max.)	Without glaucoma or OHT	With glaucoma or OHT	*p*
SCL (µm)	254 ± 53 255– (130–371)	288 ± 54 294– (210–363)	0.090
SCA (µm^2^)	4360 ± 1837 4180– (1916–8654)	6144 ± 2298 6015– (3397–9066)	**0.026**

*Mann–Whitney U test*

*SCL*, Schlemm’s canal length; *SCA*, Schlemm’s canal area; *GO*, Graves’ ophthalmopathy; *SD*, standard deviation; *OHT*, ocular hypertension

*p* < 0.05 was shown in bold

The findings related to GD duration, GO duration, and CAS in the GO group are presented in Table 4.

**Table 4:** GD duration, GO duration, and CAS in the patient group

	Mean	SD	Median	Min	Max
GD duration (months)	48	60	36	16	360
GO duration (months)	35	15	33	11	72
CAS	2	2	2	0	6

*GD*, Graves’ disease; *GO*, Graves’ ophthalmopathy; *CAS*, clinically activity score; *SD*, standard deviation

## Dıscussıon

In our study, both mean SCL and SCA were found to be significantly decreased in patients with GO compared to controls. Additionally, the mean IOP and Hertel values were higher in the GO group than in the healthy controls. In the subgroup analysis of eyes with GO, SCA was found to be higher in those with glaucoma or OHT compared to those without.

Anterior segment OCT (AS-OCT) is a valuable non-invasive tool for rapidly imaging and measuring fine structures of the anterior segment [20]. While studies evaluating SC using AS-OCT are lacking in GO eyes, various studies have examined SC in glaucomatous eyes using this technology [9, 11, 21]. Hong et al. reported that the SCA was lower in eyes with primary open-angle glaucoma compared to normal eyes, and İmamoğlu et al. found that the mean SCL was shorter and SCA was smaller in eyes with pseudoexfoliation glaucoma compared to controls [9, 11]. The correlation between SC size and outflow facility has been reported, suggesting that reduced SC size may be associated with elevated IOP [10]. Additionally, studies in cadaveric eyes have demonstrated a correlation between decreased SC size and outflow facility [22].

It has been reported that as IOP increases, the trabecular meshwork expands toward the SC lumen and concurrently causes the narrowing of the lumen [23]. Moreover, several studies have observed IOP elevation in GO eyes [6, 24]. Elevated IOP in healthy individuals has also been shown to decrease the cross-sectional area of SC [25, 26].

There are several theories regarding the mechanisms of increased IOP in thyroid-associated ophthalmopathy. These include increased mucopolysaccharide deposition in the trabecular meshwork, increased resistance to trabecular outflow, increased episcleral venous pressure resulting from orbital congestion and obstruction of venous outflow, a genetically linked predisposition to glaucoma, and restriction and compression of the globe by fibrotic and enlarged rectus muscles [4, 12, 27–31]. Additionally, O’Brien et al. reported that human thyroid glucosamine receptor 1 mRNA (NAGR1) and thyroid autoantigen (truncated actin-binding protein) in SC cells were overexpressed at > 2.5-fold compared to juxtacanalicular trabecular meshwork cells [32]. Therefore, there may be a mechanism directly related to thyroid-related receptors and proteins present in SC cells.

In our study, although SCL and SCA were found to be lower in GO eyes compared to controls, no correlation was found between SCL, SCA, and IOP. These results raise several questions. Could the decreased SC parameters in GO be due to the restriction and compression of the globe caused by the ophthalmopathy? Could the accumulation of glycosaminoglycans in the surrounding tissues, similar to the trabecular meshwork, lead to the narrowing of SC? Is there a mechanism directly related to thyroid hormones, receptors, and autoantigens present in SC cells? These questions can only be answered through further in vivo studies with a larger number of patients and controls, new in vitro studies, and investigations using cadaveric eyes.

Although there is no definitive causal relationship between the elevated IOP characteristic of thyroid-associated ophthalmopathy and primary open-angle glaucoma, these patients are often managed as glaucoma suspects [33, 34]. In our study, only 2 eyes (3.1%) of the GO group were diagnosed with glaucoma, and 8 eyes (12.5%) were diagnosed with ocular hypertension during follow-up. These patients are currently under regular follow-up at our clinic. None of the patients in the control group received a diagnosis of glaucoma or ocular hypertension.

In our study, in GO eyes, while no significant difference was observed in SCL among subgroups, SCA was found to be elevated in those with glaucoma or OHT. In previous studies, the relationship between elevated IOP and SC size [10], as well as the narrowing of SC lumen with increasing IOP [23], has been reported. In accordance with the aforementioned studies, our anticipated outcome was a reduction in the SCA. However, our findings did not align with this anticipated association. We believe that further studies including more patients with both groups in GO eyes are needed to clarify this issue.

Our study has some limitations. First, the study was conducted at a single center, resulting in a relatively small number of cases. Secondly, a more detailed evaluation of SCL and SCA based on different quadrants could have been possible by obtaining anterior segment OCT images from different quadrants. Additionally, investigating the relationship between SC parameters and thyroid receptor autoantibody levels could have been performed during the study period. Lastly, as the study was cross-sectional, changes in SC parameters over time due to the course of GD and GO could not be observed.

In conclusion, our study found that SCL and SCA were lower, and IOP was higher in GO eyes compared to controls. The findings from our study, along with future studies involving a larger number of patients, will contribute to a better understanding of the GO-IOP relationship.
